# Identification of Eps15 as Antigen Recognized by the Monoclonal Antibodies aa2 and ab52 of the Wuerzburg Hybridoma Library against *Drosophila* Brain

**DOI:** 10.1371/journal.pone.0029352

**Published:** 2011-12-19

**Authors:** Partho Halder, Yi-chun Chen, Janine Brauckhoff, Alois Hofbauer, Marie-Christine Dabauvalle, Urs Lewandrowski, Christiane Winkler, Albert Sickmann, Erich Buchner

**Affiliations:** 1 Department of Neurobiology and Genetics, Theodor-Boveri Institute, University of Wuerzburg, Wuerzburg, Germany; 2 Institute of Clinical Neurobiology, University of Wuerzburg, Wuerzburg, Germany; 3 Department of Developmental Biology, Institute of Zoology, University of Regensburg, Regensburg, Germany; 4 Division of Electron Microscopy, Theodor-Boveri Institute, University of Wuerzburg, Wuerzburg, Germany; 5 Rudolf Virchow Center, DFG Research Center for Experimental Biomedicine, University of Wuerzburg, Wuerzburg, Germany; 6 Leibniz Institut für Analytische Wissenschaften-ISAS-e.V., Dortmund, Germany; 7 Medizinisches Proteom-Center, Ruhr-Universität Bochum, Bochum, Germany; VIB & Katholieke Universiteit Leuven, Belgium

## Abstract

The Wuerzburg Hybridoma Library against the *Drosophila* brain represents a collection of around 200 monoclonal antibodies that bind to specific structures in the *Drosophila* brain. Here we describe the immunohistochemical staining patterns, the Western blot signals of one- and two-dimensional electrophoretic separation, and the mass spectrometric characterization of the target protein candidates recognized by the monoclonal antibodies aa2 and ab52 from the library. Analysis of a mutant of a candidate gene identified the *Drosophila* homolog of the Epidermal growth factor receptor Pathway Substrate clone 15 (Eps15) as the antigen for these two antibodies.

## Introduction

The generation of hybridomas was one of the milestones in modern biology [1], [2] leading to the production of monoclonal antibodies (mAbs), one of the most important tools in biology. Instead of remaining as mere tools for research, mAbs have become indispensible in therapeutics as well as diagnostics [3]. Today mAbs are important therapeutic agents for a wide variety of diseases like cancers [4], autoimmunity [5], respiratory diseases [6], infectious diseases [7] and AIDS [8]. Currently mAbs represent over 30% of all biological proteins undergoing clinical trials and are the second largest class of biodrugs after vaccines [9]–[11]. With the advent of more efficient, genetically engineered antibodies [12] this trend is expected to grow [13], [14]. The advancement of technology and the widespread applications of mAbs led to the development of alternate methods of production, like nonrodent hybridomas [15], plants [16], ascites [17] and bioreactors [18]. Soon after the production of mAbs against specific proteins, mAbs were randomly generated as ‘hybridoma libraries’ against complex protein mixtures from tissues or subcellular compartments [19]–[21]. The production of such libraries against the *Drosophila* nervous system was pioneered by the group of the late Seymour Benzer [22], [23]. One such extensive hybridoma library, generated against *Drosophila* brain homogenate is the Wuerzburg Hybridoma Library [24], [25]. MAbs from this library can be used as tools for cell-specific neuroanatomical staining [24] and, in favorable cases, for the identification of novel brain proteins by either a “candidate” or “from antibody to gene” approach. By the candidate approach the antigen of mAb nb33 which binds to pigment dispersing factor (PDF) containing neurons was identified as the PDF precursor protein (but not the PDF itself) [25]. The approach “from antibody to gene” has led to the discovery of several novel synaptic proteins. Initially, target proteins were identified by screening of cDNA expression libraries for clones expressing proteins with an epitope recognized by a given mAb. The cysteine string protein (CSP) and its gene (*Csp*) was discovered by mAb ab49 [26], the synapse-associated protein of 47 kD (SAP-47) and its gene (*Sap47*) was discovered by mAb nc46 and later shown to be also recognized at a different epitope by nb200 [25], [27]. More recently, protein purification and mass spectrometry was used for the identification of the protein Bruchpilot (BRP) and its gene (*brp*) recognized by the mAb nc82 [28], [29]. In addition to the mAbs generated against brain homogenate our hybridoma library contains mAbs 3C11 and pok13 which were generated against bacterially expressed *Drosophila* synapsin and calbindin-32, respectively [30], [31]. Besides these mAbs with known targets the library contains a large collection of mAbs which recognize different structures like body tissues (eyes, muscles, cuticula, perineureum, trachea) or cellular compartments (cell-body layers, nucleus, membranes) or small subsets of neurons, but whose target proteins are unknown [25]. Only few antibodies with unknown targets bind to synaptic neuropil. In line with our long standing research focus on synaptic proteins [26]–[31] we here describe the mAbs aa2 and ab52, their binding to synaptic neuropil of the adult brain, their immunohistochemical staining of the synaptic boutons of larval motor neurons, and the experiments leading to the identification of the homologue of Epidermal growth factor receptor Pathway Substrate clone 15 (Eps15) as the antigen they recognize in the *Drosophila* brain. Eps15 is a substrate for the tyrosine kinase activity [32] of the Epidermal Growth Factor Receptor (EGFR) and contains Ca^2+^ binding EF hands, which comprise the Eps15 homology (EH) domain [33]. Proteins containing EH domains interact with partner proteins containing the Asn-Pro-Phe (NPF) motif and play an important role in synaptic vesicle recycling and receptor endocytosis [34].

## Materials and Methods

### Fly strains

Unless indicated otherwise, Canton S (CS) was used as the wild type for all experiments. *eps15^Δ29^* mutant flies [35] were kindly provided by H. Bellen.

### Hybridoma cell culture, monoclonal antibody production

Hybridoma clones were generated as described earlier [24], [25]. For mAb production, cryopreserved cell lines were thawed and cultured, initially in 24 well Nunclon™Δ plates (Nunc) with HT medium as described earlier [25]. 50 µl of fetal bovine serum was added per well to facilitate the initial growth of the thawed cells. Growth of cells was monitored daily under an inverted microscope (Zeiss). Upon proliferation after 2–3 days, 1 ml of actively growing cell suspension was used to inoculate 5 ml HT medium in 50 ml T flasks (Greiner Bio) and further cultured for 2 days. Thereafter fresh medium was provided and after 2 more days cell density was determined by Trypan Blue (Sigma) exclusion staining of cell suspension using a Neubauer-counting chamber (GLW). When the supernatant appeared yellowish (cell density ∼10^6^ cells/ml), supernatant medium was withdrawn and centrifuged at 2000 rpm for 5 min to pellet all cells and the cell-free supernatant was tested for the presence of antibodies to find the optimal dilution for a reliable signal in western blots and/or immunohistochemistry. Upon detection of an antibody signal, the antibody producing cells were further cultured for continued antibody production until the cell density reached ∼10^6^ cells/ml. At this stage they could be either split into more flasks or used to inoculate larger flasks (250 ml, 75 cm^2^, Grenier Bio). Supernatant from larger flasks was withdrawn every 3 days.

### Characterization of the monoclonal antibodies

For the characterization of monoclonal antibodies their isotype was determined by capture ELISA using the ISO2-KT (Sigma) mouse monoclonal isotyping kit following the manufacturer's instructions. 5-Aminosalicylic acid (Sigma) was used as substrate (1 mg/ml) in 0.02 M sodium phosphate buffer (pH 6.8) with 0.01% H_2_O_2_ (v/v). Isotype of a given mAb was visually evident as development of color by the chromogenic substrate with its corresponding anti-isotype antibody. The isotype was further confirmed by immunoassay based Isoquick Strips (Envirologix) following the manufacturer's instructions. For storage of the monoclonal antibodies, suitably sized aliquots of the culture supernatant were snap frozen in liquid nitrogen and stored at −20°C. However in case of IgM antibodies, which (like IgG3) tend to aggregate upon freezing and thawing, they were stored at 4°C by adding 0.02% NaN_3_ (w/v) as antimicrobial agent.

### Immunostaining of fly heads

Cryosections of adult fly heads were made essentially as previously described [36]. Series of consecutive sections were collected on subbed glass slides (Menzel Gläser), thawed on the slide and air-dried. The slides were blocked for 2 hr at RT in a humid chamber with normal serum from the species in which the secondary antibody was generated (Vector Labs) diluted 1∶20 in 1x PBST (phosphate buffered saline, pH 7.6 with 0.1% Triton-X100). Thereafter the sections were incubated with primary mAbs (aa2 1∶2, ab52 1∶5) in PBST at 4°C overnight in a humid chamber. Excess primary mAb was removed and the slides were washed three times for 20 min each with PBST. The slides were incubated with Cy2 labeled α-mouse secondary Ab (diluted 1∶500 in PBST) and either DAPI (0.2 µg/ml) or Cy3 labeled anti-HRP (1∶500) (Jackson Immuno Res. Inc.) at room temperature for 2 hrs (anti-HRP cross-reacts with a carbohydrate epitope on *Drosophila* neuronal membranes). After washing in PBST the sections were permanently mounted with Vectashield® (Vector Labs). Optical sections were obtained by a confocal scanning microscope (Leica TCS-SP2).

### Immunostaining of motorneuron terminals

The procedure for obtaining larval nerve-muscle preparations for immuno-labeling has recently been described [37]. The ‘filets’ were blocked in 5% normal goat serum (Vector Labs) in 1x PBST for 1 hour at RT with gentle shaking. Thereafter the preparations were incubated overnight in the primary antibody (mAB aa2 diluted 1∶2, mAb ab52 (1∶2), guinea pig anti-Eps15 antiserum (kindly provide by H. Bellen) (1∶300) in PBST) at 4°C. Next day the preparations were washed in 1x PBST', once for 30 min and 4 times for 1 h each at room temperature with gentle shaking. Then they were incubated overnight in the goat-α-mouse-Alexa488 and goat-α-guinea pig Cy3 secondary antibodies (Invitrogen), both diluted 1∶500 in the blocking solution at 4°C. Next, the preparations were washed 3 times in PBST for 20 min each at room temperature with gentle shaking. The preparations were mounted in Vectashield®. Images were acquired with a confocal scanning microscope. The confocal stacks were analyzed using the Fiji package [38] based on ImageJ [39], [40].

### SDS-PAGE and Western blot

Samples were prepared in 1x LDS sample buffer (Invitrogen) and resolved using the NuPAGE® precast gel system (Invitrogen) by SDS-PAGE. In brief, samples were run on Novex® Bis-Tris 12% gels with 1x MOPS SDS running buffer (Invitrogen). Gels were transferred onto 0.45 µm nitrocellulose membrane (Protran®, Whatman) with 3 mm Chr paper (Whatman) sandwiches in a Mini Trans-Blot® (Bio-Rad) apparatus using the Towbin buffer system [41] at 100 V for 1 hr. Thereafter the membranes were stained with Ponceau S (0.1% w/v) solution (Sigma) and blocked in 5% (w/v) non-fat dry milk (Roth) in 1x TBST (10 mM Tris pH 7.6, 150 mM NaCl, 0.05% v/v Tween-20) for 2 hours at room temperature. Thereafter blots were incubated overnight in primary antibodies at suitable dilutions in 1x TBST at 4°C. The mAb aa2 was used at dilution of 1∶2 while ab52 was used at a dilution of 1∶10. Next morning, blots were washed in 1x TBST three times for 5 minutes each and then incubated for one hour with the goat-α-mouse-HRP secondary antibody (Bio-Rad) diluted at 1∶7500 in 1x TBST at room temperature. Thereafter blots were washed as earlier and developed with ECL™ (Amersham, GE) and signals were obtained as exposures on X-Ray films in the dark and developed them using developing and fixing solutions (Kodak).

### Subcellular fractionation

Adult CS flies were anesthetized with CO_2_, collected in 50 ml falcon tubes and snap frozen by immersing the tubes in liquid N_2._ Frozen flies were vigorously vortexed to separate all jointed body parts and passed through a stack of two sieves. The upper sieve with 800 µm mesh size retained thorax and abdomen while the lower sieve with 500 µm mesh size retained the heads, and smaller body parts passed through. Frozen heads from the lower sieve were collected and pulverized in a mortar-pestle, which was prechilled to −80°C. The powder was dissolved in homogenization buffer A (50 mM Tris, 150 mM NaCl, 1 mM EDTA, 1 mM EGTA final pH 7.3) supplemented with 1 tablet (per 10 ml buffer per 1 gm of fly heads) of protease inhibitors mix (Complete Mini™, Roche). The sample was thoroughly mixed to get a uniform homogenate, which was then incubated on ice for 5 minutes. Thereafter it was spun twice at 13000 rpm for 15 min each, at 4°C to pellet the exoskeleton, cell debris, nuclei (P1). The post-nuclear supernatant (S1) was re-spun in an ultracentrifuge (L8 Beckman, 60Ti rotor) at 100000 g for 1 hr at 4°C to get the cytosolic fraction as the supernatant (S2) and the total membrane fraction as the pellet (P2) which were then tested on Western blots.

### Two-dimensional electrophoresis (2DE), IEF/SDS-PAGE

Proteins from fly head homogenate were resolved by 2D electrophoresis using the Zoom® 2D (Invitrogen) setup. In brief, 100 freshly isolated CS fly heads were homogenized in 100 µl of Zoom® 2D Protein Solubilizer1 (Invitrogen) containing 1x protease inhibitors (Complete-Mini™, Roche). The homogenate was then centrifuged at 13000 rpm for 15 min at 4°C, to remove the exoskeleton, cell debris, and nuclei. 1 µl of 99% N,N-Dimethylacrylamide (DMA, Sigma) was added to the post-nuclear supernatant and incubated on a rotary shaker at room temperature for 15 min to alkylate the proteins. Thereafter 1 µl of 2 M DTT was added to quench any excess DMA and the sample was ready for loading. 25 µl of this homogenate, equivalent to 25 fly heads was mixed with strip rehydration buffer (Zoom® 2D Protein Solubilizer1, 20 mM DTT, traces of Bromophenolblue) supplemented with 0.01% (v/v) 3–10 pH ampholyte (Serva) to get a final volume of 165 µl. Immobilized pH gradient (IPG) (Zoom®, Invitrogen) strips for the range 3–10 pH were rehydrated with this sample as per the manufacturer's instructions in the Zoom® IPG Runner™ cassette overnight at 18°C. Next day the sample in the rehydrated strips was resolved by isoelectric focusing with a Zoom® Dual power supply unit (Invitrogen), while keeping the power limited to 0.1 W per strip and using the voltage regime shown in Table 1.

**Table 1:** Voltage regime for isoelectric focusing.

Step	Voltage (V)	Time (min)	Total Volthours (Vh)
1	200	20	66.7
2	450	15	112.5
3	750	15	187.5
4	750–2000	45	468.75
5	2000	30	1000

Thereafter the strips were incubated in the equilibration solution (1x NuPAGE™ LDS sample buffer with 1x NuPAGE™ Reducing Agent, both Invitrogen) and alkylation solutions (1x NuPAGE™ LDS sample buffer) with 125 mM Iodoacetamide (Sigma) for 15 minutes respectively, with gentle shaking. Thereafter the strips were loaded into the IPG well of 4–12% Bis-Tris NuPAGE™ (Invitrogen) 2D PAA gel and overlaid with agarose (0.5% w/v in 1x MOPS running buffer). The second dimension was run at 100V after which proteins were blotted from the gel on two separate membranes consecutively to get duplicate blots of the same 2D separation profile. These membranes were then incubated with the mAb aa2 and ab52 separately and developed to compare their signal profiles.

### Two-dimensional electrophoresis NEPHGE/SDS-PAGE

For separation of larger amounts of proteins, Non-Equilibrium pH Gradient gel Electrophoresis (NEPHGE) was performed according to the method of O'Farrell *et al.* [42] with some modifications. In brief, tube gels [9 M Urea, 4% acrylamide, 2.5% NP40, 5% ampholytes (Servalyte™, Serva) pH 2–11, 0.03% APS, 0.2% TEMED] of length 11 cm and diameter 3 mm, were casted overnight. Soluble (cytosolic) fraction S2 was obtained as described above and 100 µl (100 head equivalents) were precipitated with 900 µl of chilled acetone for 3 hours at −20°C. The sample was centrifuged at 10000 g for 10 min at 4°C to pellet the precipitated proteins. The supernatant was discarded, the pellet was air-dried and resuspended in 50 µl sample loading buffer 1 (9.5 M Urea, 0.5% SDS, 5% β-mercaptoethanol, 2% ampholytes pH 2–11). Upon dissolution, an equal volume of buffer 2 (9.5 M Urea, 5% NP-40, 5% β-mercaptoethanol, 2% ampholytes pH 2–11) was added. The sample was loaded on top of the tube gel and overlaid with 40 µl of overlaying solution (6 M Urea, 5% NP-40, 1% ampholytes pH 2–11). Electrophoresis was carried out in the Model 175 Tube Cell (Bio-Rad) setup at 200 V for 15 min, followed by 300 V for 30 min and finally at 400 V for 120 min. 10 mM H_3_PO_4_ and 20 mM NaOH were used as anode and cathode electrophoresis buffers respectively. As a marker for highly basic proteins, cytochrome C having a pI>11, was loaded on one of the tube gels as a control for the progress of the 1^st^ dimension. At the end of the run, the NEPHGE gel with the sample was slowly withdrawn from the glass tube, equilibrated for 20 min with the SDS sample buffer (60 mM Tris-HCl, 2% SDS, 5% β-mercaptoethanol, 10% Glycerol, pH 6.8) and overlaid with 1% agarose in SDS sample buffer on a 12% PAA gel. 10 freshly homogenized fly heads were also loaded in an adjacent lane to serve as a 1D reference to the 2D profile. Electrophoresis was carried out at 15 mA for 16 hr. The tube gel with cytochrome C was cut into 0.5 cm pieces and each piece was incubated overnight with 3 ml dH_2_O at 4°C such that the pH along the length of the gel could be measured next morning.

### Partial blot and silver staining of NEPHGE gel

After 2DE, the gel was blotted for 20 minutes using the Towbin transfer buffer system at 2 mA/cm^2^ of gel area in a PerfectBlue™ (peqLAB) semi-dry blotting apparatus. By this procedure only part of the protein content of the gel was transferred onto the membrane, while the rest was retained in the gel, which was then silver stained for MS compatibility as described earlier [43]. The blot was blocked as described above and then incubated with primary antibody (ab52, diluted 1∶10) and developed as described above. Overnight exposure was done to get a strong signal for the antigen along with weak non-specific signals on the blot, which would serve as landmarks for comparison with the silver-stained gel. Images of the over-exposed blot and the silver-stained gel were digitally superimposed with Photoshop (Adobe) to pinpoint the protein spot in the silver stained gel that corresponded to the signal in the Western blot. This spot was then excised and analyzed by mass spectrometry as described below.

### Immunoprecipitation of the antigen for mAb aa2

3000 fly heads were homogenized in 1 ml of homogenization buffer (50 mM Tris, 100 mM NaCl, 10% glycerol, 1% Triton X-100, 1.5 mM MgCl_2_, 0.01% β-mercaptoethanol, protease inhibitors as in buffer-A, final pH 7.5), centrifuged at 14000 rpm, 4°C for 10 min to yield the supernatant S1. 600 µl of protein-G agarose beads (Roche) were washed in homogenization buffer and incubated in 8 ml of undiluted mAb aa2 supernatant (neutralized to pH 7.5 with 1 M Na_2_HPO_4_) for 3 h at 4°C with gentle mixing, followed by washing and incubation with supernatant S1 for 3 h at 4°C. The mixture was centrifuged (1500 rpm, 4°C, 2 min), the pellet was washed with 1 ml homogenization buffer, and 60 µl of 5x Lämmli buffer was added to the beads. The sample was heat denatured and loaded in two gels and resolved in parallel by SDS-PAGE. One gel was stained by Coomassie [44] while the other was blotted. The blot was blocked as described earlier and incubated with the primary antibody aa2 (1∶2), followed by development as described above. Superimposition of the stained gel and blot images allowed the identification of the proteins bands in the stained gel that corresponded to the Western blot signal. The specific bands were excised and analyzed by mass spectrometry as described below.

### Mass spectrometric (MS) analysis

The protein spot from the silver stained NEPHGE gel, corresponding to the mAb ab52 Western signal, and the Coomassie-stained immunoprecipitated proteins in the 1-D SDS gel corresponding to the mAb aa2 Western signal were excised and the proteins were reduced, carbamidomethylated, and digested by trypsin followed by MS analysis as described earlier [25]. For data evaluation, raw-data was converted to Mascot-mgf files using ProteomeWizard (http://proteowizard.sourceforge.net/).Searches were conducted against a subset of the Swissprot database (www.uniprot.org) containing 35373 sequences (5th May 2011). Mascot (v2.2) was used as search engine with Mascot Daemon support (v2.2.0) with the following parameters: Trypsin was set as protease with one miscleavage site allowed, precursor and fragment ion tolerance was 0.5 Da, carbamidomethylation (C) was chosen as fixed and oxidation (M) as variable modification. Peptides with p<0.01 and scores above 38 were considered for subsequent manual validation.

## Results and Discussion

### Characterization of the mAb isotypes

aa2 was found to be an IgG1, while ab52 was found to be an IgM. The fact that aa2 and ab52 are of different isotypes indicates that they are produced by two distinct hybridoma cell lines and hence not subclones from a common parent hybridoma cell. Both mAbs had the kappa (κ) type of light chain.

### Staining pattern of the mAbs aa2 and ab52

**Figure 1:**
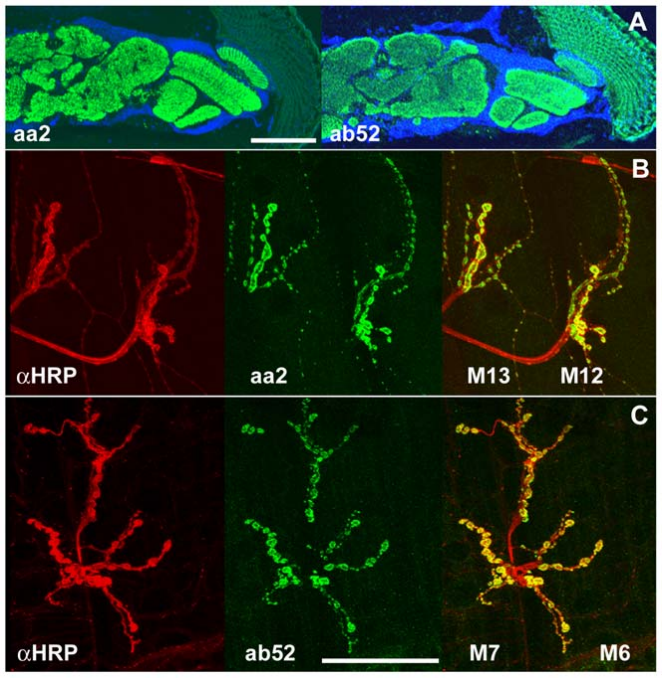
Staining patterns of mAbs aa2 and ab52. (A) Cryosections of an adult fly head were probed with mAb aa2 (left) and mAb ab52 (right). Both antibodies stain all synaptic neuropil (green) but not the surrounding the cell body layer whose nuclei are stained with DAPI (blue). (B, C) Synaptic boutons of larval motor neuron terminals stained with anti-HRP (left, red) and mAbs aa2 (B, middle, green), or ab52 (C, middle, green). The overlays in the right column demonstrate that the epitopes recognized by both mAbs are present in all boutons (here shown for muscles M12/13 (B) and muscles M6/7 (C)) but not in the axons. Scale bars in A: 100 µm; in C for B and C: 50 µm)

On cryosections of adult heads these two antibodies equally stain all synaptic neuropil as shown in Fig. 1A. In larval nerve-muscle preparations both antibodies stain all synaptic boutons (Fig. 1B,C).

### Migration pattern of antigens recognized by aa2 and ab52 on 1DE

**Figure 2:**
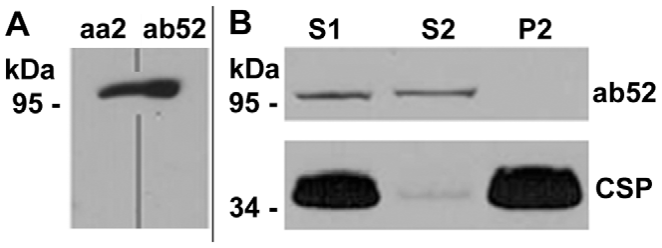
mAbs aa2 and ab52 recognize soluble proteins at identical M_r_ on 1D Western blot. (A) Blot of a single SDS gel electrophoresis lane loaded with homogenate from 2 wild-type (CS) heads. The blotted membrane was vertically cut in two halves; one was developed with mAb aa2 (left), the other with mAb ab52 (right). Signals at identical M_r_ suggests that both mAbs probably recognize the same antigen. (B) Western blot of subcellular fractions of wild-type (CS) fly heads showing that the protein detected by mAb ab52 is exclusively present in the cytosolic supernatant. S1  = postnuclear supernatant; S2 =  cytosolic fraction; P2 =  total membrane fraction (10 head equivalents loaded per lane). The synaptic vesicle protein CSP recognized by the mAb ab49 was used as a marker for the total membrane fraction.

In Western blots of freshly homogenized CS fly heads, both antibodies produce a single signal around 100 kDa. To test for identical migration properties of the recognized antigens, proteins from 2 freshly homogenized CS fly heads were resolved by SDS-PAGE, followed by Western blotting and then the blot of a single lane was vertically cut into two halves. One half of the lane was incubated with mAb aa2 and the other with mAb ab52, both halves were separately washed, incubated with secondary antibody, washed again and then developed together (Fig. 2A). The developed blots suggest that the antigens recognized by the two mAbs have the same M_r_ and hence may be the same protein.

### Subcellular fractionation of ab52 antigen

Upon subcellular fractionation as described in Materials and Methods, the mAb ab52 antigen remained in the cytosolic supernatant (S2), indicating that it is soluble under the conditions of homogenization (Fig 2B). CSP (cysteine string protein) detected by mAb ab49 [26], remains in the pellet, which represents the total membrane fraction, thus demonstrating the effectiveness of the fractionation.

### Migration pattern of antigens recognized by aa2 and ab52 on 2DE

**Figure 3:**
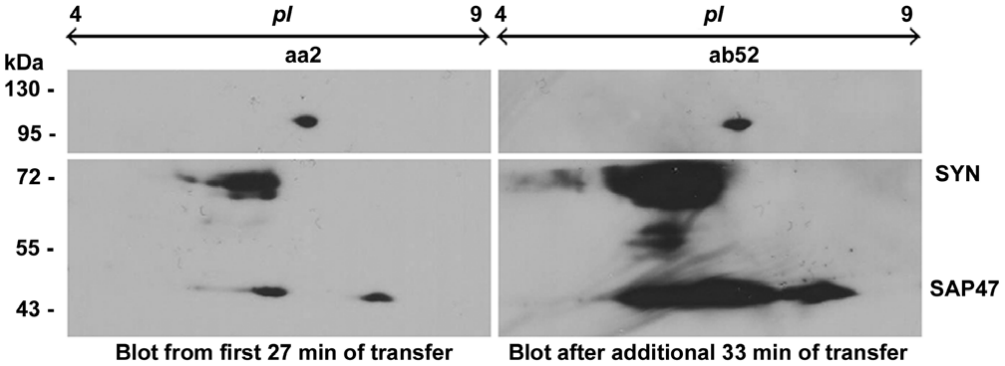
aa2 and ab52 produce signals at identical M_r_ and pI on 2D Western blot profile. Two sequential Western blots from a single 2DE gel loaded with sample equivalent to 25 fly heads. The two membranes were cut along the horizontal white line, the upper parts were developed with mAbs aa2 (left, dilution 1∶2) or ab52 (right, dilution 1∶10), the lower parts were stained with mAbs 3C11 (anti-SYN, 1∶100) and nc46 (anti-SAP47, 1∶200) as controls for both blots.

Since aa2 and ab52 seemed to recognize the same antigen on 1DE and since the antigen recognized by ab52 was found to be a soluble, cytosolic protein, we used 2DE to resolve this protein as a distinct spot and compared the Western blots signals of the 2DE profile for both mAbs aa2 and ab52. CS fly head homogenates were resolved by 2DE (IEF/SDS-PAGE) followed by partial Western blot of the same gel consecutively on two separate membranes to get duplicate blots of the same 2DE separation profile. Development of the blots incubated with aa2 and ab52 separately, revealed signals for both antibodies as a single, distinct spot with identical patterns having M_r_ ∼100 kDa in the pI range 6–7 as shown in Fig 3. This further indicated that both mAbs indeed recognize the same antigen.

### Enrichment of the antigen of mAb aa2 by immunoprecipitation

**Figure 4:**
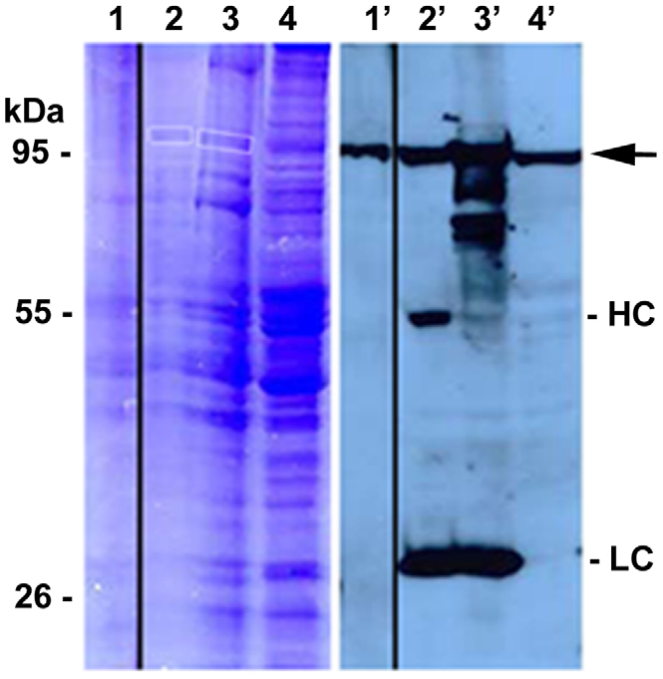
Identification of mAb aa2 antigen by comparison of a Coomassie stained gel and a Western blot. Coomassie-blue stained gel (left) and Western blot of a gel run in parallel (right). Loaded was: supernatant S1 (3 head equivalents, lanes 1, 1′), immunoprecipitation of S1 with mAb aa2 and protein-G beads (∼80 head equivalents, lanes 2, 2′ or ∼800 head equivalents, lanes 3, 3′), and 3 heads homogenized in Lämmli buffer (lanes 4, 4′). The blot was developed with mAb aa2. LC, light chain, HC, heavy chain of mAb aa2. The boxed bands of the gel were cut out and subjected to MS analysis.

mAb aa2 was used to enrich the target antigen it recognized by immunoprecipitation (IP) to facilitate its identification by MS. Homogenized fly heads were subjected to immunoprecipitation using serum-free supernatant and protein G beads as described in Materials and Methods. Identical aliquots of the S1 input to the IP, of the proteins eluted from the beads by SDS buffer, and of homogenate from 3 fresh heads were loaded in two gels, one was Coomassie stained and one was blotted and developed with mAb aa2. Among numerous bands in the Coomassie-stained gel, one band appeared to correspond to the mAb aa2 signal in the Western blot (boxed in Fig. 4). Note that in lane 3′ compared to lane 2′ of the gel a tenfold higher amount of protein was loaded, leading to the recognition of proteolytic degradation products of the aa2 antigen in the blot lane 3′.

### Enrichment of the antigen of mAb ab52 by NEPHGE followed by SDS PAGE

**Figure 5:**
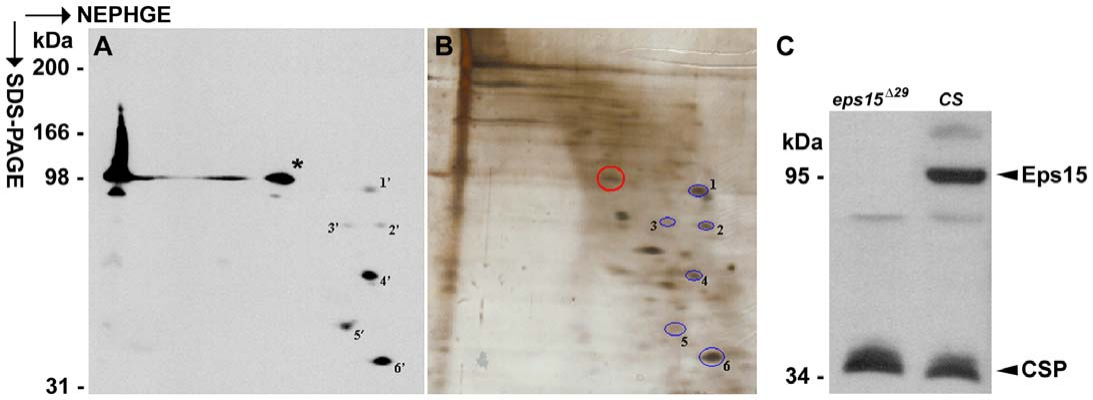
Enrichment of the ab52 antigen for MS by NEPHGE/SDS-PAGE and its final identification as Eps15. (A) Western blot developed with mAb ab52 after partial transfer of proteins from the gel shown in (B). The signal from the mAb ab52 can be clearly seen as a distinct spot ∼100 kDa (asterisk), with a corresponding strong signal in the 1D lane loaded with 10 freshly homogenized fly heads. Non-specific signals were numbered as landmarks 1′–6′. (B) Silver stained NEPHGE gel with protein spots corresponding to the non-specific Western signals of (A) numbered 1–6 and the spot corresponding to the Western signal from mAb ab52 (encircled). (C) Western Blot developed with mAbs ab52 and ab49 showing that in the Eps15 null mutant *eps15^Δ29^* the ∼100 kDa signal characteristic for ab52 in the wild type (CS) is absent. CSP recognized by mAb ab49 was used as a loading control.

mAb ab52 was found to be an IgM, making its application in IP for enrichment of its target antigen unsuitable. However the soluble nature of the antigen allowed us to resolve it as a distinct spot by 2DE. Thus the cytosolic supernatant (S2) was subjected to Non-Equilibrium pH Gradient gel Electrophoresis (NEPHGE) followed by SDS-PAGE as described in **Methods.** Western blotting (Fig. 5A) was done for only 20 min, to transfer only part of the total protein content of the gel onto the membrane, while retaining the rest of it in the gel, which was later visualized by MS-compatible silver staining (Fig 5B). The blot was incubated with mAb ab52 and developed with a long exposure of 2 hours to obtain the specific signal for ab52 and in addition, some non-specific spots (Fig. 5A). The non-specific spots on the Western blot were numbered 1′–6′ and their corresponding spots on the gel were numbered 1–6. These pairs of spots were used to align the blot to the silver-stained gel and thus pinpoint the silver-stained spot (circled in Fig. 5B) corresponding to the mAb ab52 antigen signal in the Western blot.

### Mass spectrometric identification of the antigen candidates

The bands cut out from the gel of the IP experiment (boxed in Fig. 4) were analyzed by mass spectrometry after proteolytic digestion and signals were matched. As is common for 1D-PAGE separations, several different *Drosophila* proteins were identified in each band, of which 12 had a score>300 (Table 2). Three of the identified proteins are known components of the peri-active zone of *Drosophila* synapses and thus in view of the localization data are preferred candidates: Eps15, shibire (dynamin) and α-adaptin. Furthermore, Dap160 was identified as a low scoring but significant component of lane 3 (score 83, 3 peptides matched). For a detailed list of all identified proteins please refer to table S1. Dap160/intersectin is a prominent binding partner of Eps15 [35]. This suggests that one of these proteins could be the desired antigen while the other three may have been co-immunoprecipitated and, due to similar molecular weights, enriched in the cut-out gel pieces.

**Table 2:** Proteins with cumulative Mascot scores>300 identified in the gel pieces boxed in lane 2 and 3 of Fig. 4.

Accession	Protein	MW	Cumulative Mascot	Sequence coverage
			scoring lane 2/3	lane 2/3
Q9XTL9	Glycogen phosphorylase	97334	746/916	22/24
P27619	Dynamin *	98147	678/249	16/6
Q9W0E4	Puromycin sensitive aminopeptidase *	99891	612/−	11/−
A4V310	Cheerio, Filamin *	93171	594/364	11/8
Q7KN62	Transitional endoplasmic reticulum ATPase TER94	89545	423/704	11/13
P91926	AP-2 complex subunit alpha/alpha adaptin*	106352	411/−	9/−
Q8MMD3	Epidermal growth factor receptor pathway substrate clone 15*	119761	348/593	9/10
P13060	Elongation factor 2 *	95424	310/−	9/−
Q9VAY2	Glycoprotein 93	90296	258/371	8/10
Q9VUC1	Hsc70Cb, isoform A	89016	257/471	9/12
Q9V9U3	CG1910 *	51670	530/−	23/−
P41073	Zinc finger protein on ecdysone puffs	78570	136/386	6/11

Protein identifications marked with asterisks (*) featured several different accession entries which belonged to the same protein but isoforms were not resolvable. Accessions are given in Swiss-Prot format.

**Figure 6:**
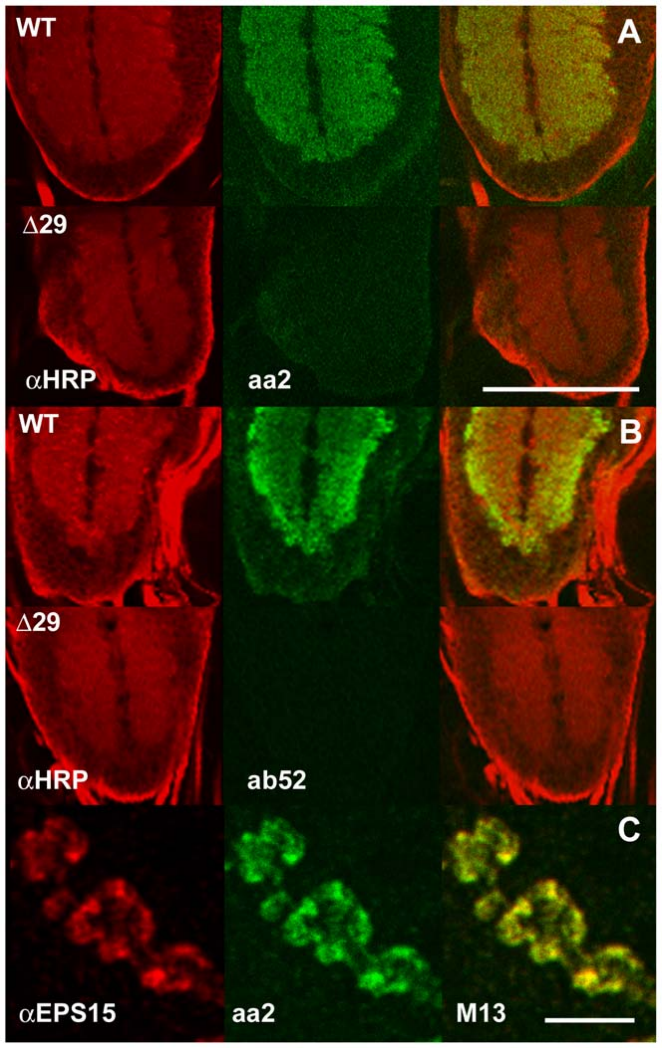
Verification of the prime candidate from MS, EPS15, as the antigen recognized by mAbs aa2 and ab52. Immunohistochemical staining of larval synaptic neuropil with mAbs aa2 (A) and ab52 (B) is present in wild type (WT) but absent in *eps15^Δ29^* null mutants (Δ29) and perfectly matches the distribution of Eps15 in synaptic boutons, here shown on muscle M13 (C). Scale bar in A for A and B: 100 µm; in C: 5 µm.

The gel piece analyzed from the NEPHGE 2D gel (encircled in Fig. 5B) contained proteins which could be matched to three entries of the of the *Drosophila* proteome (Table 3). Since Hsc70Cb and Bicaudal D have not been reported to match to the synaptic neuropil or the synaptic localization of the mAb ab52 antigen these proteins presumably are false positive hits leaving Eps15 as a strong candidate. We next demonstrated that a Western blot of an adult *eps15^Δ29^* null mutant escaper produced no signal with mAb ab52 but normal SAP47 loading control signals (Fig. 5C) and that *eps15^Δ29^* null mutant larvae showed no synaptic neuropil staining with mAbs aa2 or ab52 (Fig. 6A,B). Note that with mAb ab52 there is a gradient of staining intensity from the periphery to the center of the neuropil, indicating the (large) IgM penetrates whole mounts less easily than the IgG aa2. Finally, we show that the immunohistochemical signals generated within synaptic boutons of larval nerve-muscle preparations by mAb aa2 exactly match the signals generated by anti-Eps15 antiserum (Fig. 6C).

**Table 3:** Proteins identified in the gel piece encircled in Fig. 5B.

Accession	Protein	MW	Cumulative Mascot scoring	Sequence Coverage
Q8MMD3	Epidermal growth factor receptor pathway substrate clone 15*	119761	1039	35
Q9VUC1	Hsc70Cb *	89016	483	13
P16568	Protein Bicaudal D	89127	115	3

We thus conclude that mAbs aa2 and ab52 of the Würzburg Hybridoma Library indeed recognize the same protein, Eps15 of *Drosophila*, a protein of the peri-active zone required for normal synaptic bouton development and synaptic vesicle recycling [34], [35], [45], [46]. The two mAbs are of different isotypes produced by two distinct hybridoma lines. mAb aa2 being an IgG1 is more suitable for applications like whole mount stainings and immunoprecipitation (IgMs usually do not bind to protein-A or –G), while for immunostainings on sections or motor neuron terminals and Western blots mAb ab52 (being an IgM) is also suitable.

## Supporting Information

Table S1 **Extended MS results for boxed bands from** **Figure 4** **lane 2 and 3 and for encircled spot of 2DE gel in** **Figure 5B.**
(XLS)
